# Supplementary material for: Leveraging perturbations to infer the population dynamics of human rhinovirus and interaction of influenza A virus
Source: PLoS Comput Biol. 2026 Sep 11;22(9):e1014784. doi: 10.1371/journal.pcbi.1014784 (PMC13585339; doi:10.1371/journal.pcbi.1014784)
Supplement: Supplementary file 2 [file pcbi.1014784.s002.pdf]

Peer Review

PLOS Computational Biology

Manuscript No. PCOMPBIOL-D-26-00664

May 20, 2026

Title: Leveraging perturbations to infer the population dynamics of human rhinovirus and interaction of influenza A virus

Summary

This paper investigates rhinovirus (RV) population dynamics and tests whether influenza A virus (IAV) exerts viral interference on RV at the population level. The authors develop a Bayesian SIRS model fitted to US and Canadian surveillance data spanning the COVID-19 pandemic, treating non-pharmaceutical interventions (NPIs) as a large-scale natural experiment. They find that the surveillance data do not support a detectable IAV effect on RV transmission at the population level. They also demonstrate via simulation that exogenous perturbations are critical for disentangling pathogen interaction from seasonality, a contribution with broad methodological relevance.

This is a solid, well-executed study addressing an important and under-explored question in respiratory virus epidemiology. The methodological contribution to using perturbation-based inference to identify pathogen interactions that are invisible in stationary endemic dynamics is valuable and likely to be of broad utility. The negative finding is carefully hedged and is a meaningful contribution to the literature on RV–IAV co-circulation. Some comments are listed below that I believe should be addressed before publication.

Comments

Major comments

1. The null result may reflect model limitations rather than biology

The negative finding is based on the flexible seasonal transmission parametrization: 52 free weekly β values modelled as a cyclical random walk. With these many degrees of freedom in $\beta(t)$, the model can absorb IAV-driven suppression of RV into the seasonal forcing term, precisely because IAV peaks occur at a consistent annual time. The authors acknowledge this risk briefly (Fig. S13 and Discussion), but do not quantify how much attenuation of $\hat{\phi}$ this flexibility could cause. A sensitivity analysis constraining σ_β (the random-walk scale parameter) across a range of values, and reporting the resulting posteriors of ϕ, would strengthen the conclusion. In its current form, the reader cannot rule out that the flexible $\beta(t)$ is the primary reason viral interaction goes undetected.

2. Asymmetric treatment of interaction direction

The paper focuses on the direction IAV $\rightarrow$ RV, but some cited studies suggest the interaction is bidirectional or even RV $\rightarrow$ IAV. The authors justify their focus by stating that fitting IAV dynamics "poorly captured the data," but this is mentioned only briefly. A supplementary attempt at modelling the reverse direction, even if ultimately abandoned, would address a natural reviewer concern and contextualize the work more completely.

3. Google Mobility as NPI proxy

The mobility metric is not available before February 2020 and after October 2022, which requires assumption $c = 0$ outside of this range. Post-October 2022, as NPIs were relaxed heterogeneously across regions and new behavioural norms stabilized at non-baseline levels, setting $c = 0$ may introduce systematic errors in estimates of the effective reproduction number during the rebound period, exactly the period used as evidence against interaction. The sensitivity of key results to this boundary assumption could be explored, for example by allowing c to take a non-zero constant after 2022 and comparing parameter posteriors.

Minor comments

4. Unexplained geographic asymmetry in $\hat{\phi}$

The observation that $\hat{\phi}$ estimates are more negative for US locations and more positive for Canadian ones is puzzling and is flagged but not explored. Could this reflect differences in surveillance systems, testing patterns, or the timing of IAV rebounds (earlier and smaller in the US)? The current sentence-long mention leaves the reader without guidance.

5. Biological plausibility of the lag analysis

The lagged IAV incidence analysis considers lags of 1, 3, and 5 weeks. The authors note that potential interaction is expected to be primarily mediated by innate immunity, but do not justify the upper bound of 5 weeks. Given that interferon responses typically resolve within days to a week, a 5-week lag seems biologically long and can conflate immunological and epidemiological timescales. The authors could either cite immunological evidence supporting the range considered or tighten the biological rationale.

6. Upper-end R_0 estimates

The estimated R_0 range (1.15–3.35 for the US) is wide, and the upper values seem high for a common cold virus. While the overall range is noted as consistent with the literature, these specific outlying estimates are not discussed. Are they biologically plausible, or do they reflect identifiability challenges at the regional scale? Some commentary could be valuable.

7. Links in bibliography

I suggest moving the links on lines 377 and 378 in the bibliography instead of having them in the main text.

9. Repeating info in Discussion

The Discussion, while thorough, is somewhat long. The two paragraphs on RV resilience (pages 8–9) repeat parts from the Introduction and could be shortened without loss.

10. Further explanation of Figure 1

The cross-wavelet transform panels in Figure 1 may be difficult for readers less familiar with wavelet analysis, so a brief plain-language interpretation in the caption (beyond the current arrow-direction key) would help.
