# Supplementary material for: Leveraging perturbations to infer the population dynamics of human rhinovirus and interaction of influenza A virus
Source: PLoS Comput Biol. 2026 Sep 11;22(9):e1014784. doi: 10.1371/journal.pcbi.1014784 (PMC13585339; doi:10.1371/journal.pcbi.1014784)
Supplement: Supplementary file 3 [file pcbi.1014784.s004.pdf]

Dear Editor,

We thank you for allowing us to resubmit a revised version of our work. We have now revised our manuscript to account for all the points raised by the reviewers (all the changes are highlighted in orange) and we answer all their comments below.

Associate Editor Comment:

The three reviewers agree that the paper makes a strong methodological contribution, but identify a number of areas where the implementation, justification of modelling assumptions, and writing need improvement. Please ensure you address all reviewer comments in a revised version.

We thank you for these positive comments. We have addressed all reviewer comments in a revised version of our manuscript and we answer all their comments below.

Reviewers' Comments to the Authors:

Referee: 1

This paper investigates rhinovirus (RV) population dynamics and tests whether influenza A virus (IAV) exerts viral interference on RV at the population level. The authors develop a Bayesian SIRS model fitted to US and Canadian surveillance data spanning the COVID-19 pandemic, treating non-pharmaceutical interventions (NPIs) as a large-scale natural experiment. They find that the surveillance data do not support a detectable IAV effect on RV transmission at the population level. They also demonstrate via simulation that exogenous perturbations are critical for disentangling pathogen interaction from seasonality, a contribution with broad methodological relevance.

This is a solid, well-executed study addressing an important and under-explored question in respiratory virus epidemiology. The methodological contribution to using perturbation-based inference to identify pathogen interactions that are invisible in stationary endemic dynamics is valuable and likely to be of broad utility. The negative finding is carefully hedged and is a meaningful contribution to the literature on RV–IAV co-circulation. Some comments are listed below that I believe should be addressed before publication.

Thank you very much for your positive and useful comments, as well as your work on our manuscript. We detail below how we took into account your comments in our revision.

Major comments

1. The null result may reflect model limitations rather than biology
The negative finding is based on the flexible seasonal transmission parametrization: 52 free weekly β values modelled as a cyclical random walk. With these many degrees of freedom in $\beta(t)$, the model can absorb IAV-driven suppression of RV into the seasonal forcing term, precisely because IAV peaks occur at a consistent annual time. The authors acknowledge this risk briefly (Fig. S13 and Discussion), but do not quantify how much attenuation of $\hat{\phi}$ this flexibility could cause. A sensitivity analysis constraining σ_β (the random-walk scale parameter) across a range of values, and reporting the resulting posteriors of ϕ, would strengthen the conclusion. In its current form, the reader cannot rule out that

the flexible $\beta(t)$ is the primary reason viral interaction goes undetected.

We agree that a flexible seasonal transmission term can absorb IAV-driven suppression of RV in the case of stable periodic dynamics. This is precisely why our analysis exploits the perturbations induced by NPIs which disrupted the usual seasonal pattern between IAV and RV and therefore may provide additional information to tease apart seasonal forcing from potential viral interference. While the simulation study illustrates this point, we did not indeed quantify to which extent $\hat{\phi}$ could be affected by this flexibility, and your suggestion is a very good idea. We thus performed the proposed sensitivity analysis by fixing the random-walk scale parameter σ_β at 0.01, 0.05, 0.1, and 0.25 (estimated values of σ_β typically lie between $\approx 0.04 - 0.1$), and refitted the model to national-level data. The inferred posterior distribution of ϕ remained extremely robust across these values (Fig. S19, and Results p 7, ln 231-232).

2. Asymmetric treatment of interaction direction
The paper focuses on the direction IAV $\rightarrow$ RV, but some cited studies suggest the interaction is bidirectional or even RV $\rightarrow$ IAV. The authors justify their focus by stating that fitting IAV dynamics "poorly captured the data", but this is mentioned only briefly. A supplementary attempt at modelling the reverse direction, even if ultimately abandoned, would address a natural reviewer concern and contextualize the work more completely.

We indeed previously attempted to apply a similar framework to IAV (using RV/EV incidence as an external input), but found that it poorly captured the observed IAV dynamics, including post-pandemic rebounds. We focused here on the population dynamics of RV, which ultimately motivated the decision to model only viral interference from IAV to RV. We emphasize that treating IAV time series as an external input does not imply that RV cannot also influence IAV dynamics (Discussion p 9, ln 283), but developing, calibrating, and validating a tailored model to IAV dynamics would require substantial additional work and is slightly out of scope for this work. In particular, IAV dynamics are likely influenced by additional factors, including antigenic evolution and subtype interactions (H3 vs. H1, and IBV) that are difficult to model, but which might potentially need to be incorporated into a more realistic model of IAV transmission (Discussion p 11-12, ln 399-400). This is also one of the reasons that prevented us to fit a two-pathogen model (Discussion p 10, ln 343-350).

3. Google Mobility as NPI proxy
The mobility metric is not available before February 2020 and after October 2022, which requires assumption $c = 0$ outside of this range. Post-October 2022, as NPIs were relaxed heterogeneously across regions and new behavioural norms stabilized at non-baseline levels, setting $c = 0$ may introduce systematic errors in estimates of the effective reproduction number during the rebound period, exactly the period used as evidence against interaction. The sensitivity of key results to this boundary assumption could be explored, for example by allowing c to take a non-zero constant after 2022 and comparing parameter posteriors.

This is a good point. We agree and thus refitted the model across all locations while estimating an additional transmission multiplier (η) for the whole period after October 2022 to capture a potential shift in the behavioral baseline after Google mobility data were no longer available. We examined how this affected the posterior distributions of viral interaction, seasonal transmission and mean duration of immune protection (see Fig. S22, and Results p 8, ln 248-250). Estimated values of this transmission multiplier η were close to 1 (i.e., similar to pre-pandemic baseline) and had little impact on the estimates of the other parameters.

Minor comments

4. Unexplained geographic asymmetry in $\hat{\phi}$
The observation that $\hat{\phi}$ estimates are more negative for US locations and more positive for Canadian ones is puzzling and is flagged but not explored. Could this reflect differences in surveillance systems, testing patterns, or the timing of IAV rebounds (earlier and smaller in the US)? The current sentence-long mention leaves the reader without guidance.

We agree. The different surveillance systems and the usually earlier timing of IAV rebounds in US locations appear to be the two main differences between the two countries. It is however difficult to assess precisely their impact on the estimation process. These differences were obtained when fitting the model independently to each location. We explored how fitting the model simultaneously to all regions/provinces impacted the results by constraining ϕ to be shared across locations within a given country (US vs. Canada) and the rate of immune waning ω across all locations (Supporting information §S3). However, note that this approach assumes that relative differences in IAV incidence reflect true differences in viral circulation between locations rather than spatial heterogeneity in reporting effort. New estimates $\hat{\phi}$ are centered around 0 for both the US and Canada, with no difference in sign, although the Canadian estimate has a very wide CrI. The fact that these differences do not persist when fitting jointly across locations seems to suggest that they are more likely to reflect noise in the data than robust country-level differences. We included this analysis in the revised manuscript (Results p 7, ln 239-248, and Fig. S21); we also agree that the original sentence in the Discussion was not helpful and have removed it.

5. Biological plausibility of the lag analysis
The lagged IAV incidence analysis considers lags of 1, 3, and 5 weeks. The authors note that potential interaction is expected to be primarily mediated by innate immunity, but do not justify the upper bound of 5 weeks. Given that interferon responses typically resolve within days to a week, a 5-week lag seems biologically long and can conflate immunological and epidemiological timescales. The authors could either cite immunological evidence supporting the range considered or tighten the biological rationale.

That's right, if the potential interaction is primarily mediated by interferon responses, any protective effect would be expected to arise rapidly and be only short-lived (typically on the order of days to a week). Consequently, the analyses using current IAV incidence and a 1-week lag are the most biologically motivated. The longer lags (3 and 5 weeks) were included as more exploratory analyses to assess whether any association persisted beyond the expected duration of innate immune protection. In particular, our model is intentionally phenomenological, so it does not make any assumptions about what the mechanisms may be, and ecological interaction (as opposed to immune-mediated interaction (Bhattacharyya et al. 2015)), such as changes in contact rates after a primary infection, could last more than a week. We clarified this in the main text (Materials and Methods p 15, ln 510-514, Results p 5, ln 144-151, and Discussion p 10, ln 356-360). One limitation would be the potential double counting of hosts that have been reinfected during the lagged period, but this proportion should still remain small in a 5-week window.

6. Upper-end R_0 estimates
The estimated R_0 range (1.15–3.35 for the US) is wide, and the upper values seem high for a common cold virus. While the overall range is noted as consistent with the literature, these specific outlying estimates are not discussed. Are they biologically plausible, or do they reflect identifiability challenges

at the regional scale? Some commentary could be valuable.

Values reported in the literature are also quite heterogeneous (typically 1.2-2.7) and are setting-specific, so that estimates around 3 remain close to the upper bound of prior estimates. Our range also reflects the estimation of seasonal variation in transmission (Fig. 5A, C), rather than a classic single point estimate – averaged over the year, mean values range here from 1.16 to 2.04 in the United States and from 1.24 to 1.42 in Canada (Results p 8, ln 266-268). While some of the highest values (particularly for HHS region 2) are more likely to reflect difficulty in parameter estimation (also associated with wider CrIs), these estimates remain within or close to the range of biologically plausible values reported in the literature.

7. Links in bibliography
I suggest moving the links on lines 377 and 378 in the bibliography instead of having them in the main text.

Sure, we moved these links to the bibliography.

9. Repeating info in Discussion
The Discussion, while thorough, is somewhat long. The two paragraphs on RV resilience (pages 8–9) repeat parts from the Introduction and could be shortened without loss.

Thank you. We have shortened (and merged) the two paragraphs of the Discussion on RV resilience.

10. Further explanation of Figure 1
The cross-wavelet transform panels in Figure 1 may be difficult for readers less familiar with wavelet analysis, so a brief plain-language interpretation in the caption (beyond the current arrow-direction key) would help.

Good point, we expanded the figure caption to include an interpretation of the cross-wavelet transform panel.

Referee: 2

Thank you for the opportunity to review the manuscript. This work investigates the population-level interactions between respiratory viruses using long-term surveillance data and mechanistic modeling approaches. The authors demonstrate that exogenous epidemiological perturbations, such as non-pharmaceutical interventions, can help disentangle pathogen-pathogen interactions from confounding seasonal effects. In particular, the study finds no conclusive evidence that influenza A virus substantially alters rhinovirus dynamics at the population level. Overall, the work addresses an interesting question in infectious disease epidemiology and contributes to the understanding of respiratory virus interactions and seasonality. However, I do have the following concerns.

Thank you very much for your positive and useful comments, as well as your work on our manuscript. We detail below how we took into account your comments in our revision.

1. I am therefore not fully convinced that the current framework is sufficient to assess viral interference. Conceptually, viral interference refers to interactions between viruses within a host that alter the

infection outcome of at least one virus at the individual level. These within-host interactions subsequently shape population-level transmission dynamics and should be evaluated while accounting for key population characteristics, including immunity and co-circulation patterns. In this context, a multi-strain modeling framework may provide a more appropriate approach to address the research questions.

We agree that viral interference is fundamentally a within-host mechanism that can shape pathogen transmission at the population level through epidemiological feedback. A multi-pathogen model, such as that in (Nickbakhsh et al. 2019) (accounting for immunity and co-circulation patterns, including co-infections), may provide a more satisfying mechanistic description, but is far too complex to be fitted to such incidence time series alone. Instead, our approach is intentionally phenomenological and can integrate within-host viral interference (immunological) mechanisms but also ecological mechanisms of interactions, such as changes in contact rates after a primary infection (Bhattacharyya et al. 2015). Our model is intentionally simple and does not aim to explicitly resolve within-host interactions; instead, it provides an effective description of observed incidence dynamics and allows us to probe for epidemiological signatures consistent with altered transmission patterns, as well as to test the limitations of assessing interactions based on aggregated incidence data alone. Besides, using IAV time series as an external input present some advantages compared to a multi-strain modeling framework because surveillance time series may contain richer variability and signal capturing the complex dynamics of IAV (including antigenic variation, subtype interference, external seeding, pandemic perturbation) that could be lost if not adequately captured by the two-pathogen model. Our framework preserves this empirical variability, which is noisier (though we smoothed IAV data) but may also be more informative for inferring the potential effect of IAV on RV dynamics. We have clarified these different points in the revised manuscript (Discussion p 10, ln 343-360) and we now cite (Shrestha et al. 2013) that similarly used influenza time series as a covariate.

2. In the introduction, the hypotheses were clearly laid out for the bi-directional viral interference between RV and influenza. However, the model authors only considered viral interference from IAV to RV.

Yes, although we would like to nuance this a little. The bi-directional viral interference between RV and IAV is not very well established in the literature. Few cited studies actually provide support for a bi-directional interaction, and most report either a unidirectional association (RV $\rightarrow$ IAV or IAV $\rightarrow$ RV), or report negative correlations between the two viruses, which is difficult to attribute to a specific direction (or both). In the meantime, we have also added another reference and correspondence that support viral interference (solely) from IAV to RV (Introduction, p 3, ln 72-75). The focus of this work is on the population dynamics of RV, which ultimately motivates the decision to model only viral interference from IAV to RV, but this does not preclude the possibility that RV circulation may also influence IAV dynamics (Discussion p 9, ln 283). Previous attempts to apply a similar framework to IAV (using RV time series as an external input) poorly fitted the data, notably post-pandemic rebounds. We feel that developing, calibrating, and validating a model tailored to IAV would likely require substantial additional work and is slightly out of scope for this study.

3. I am curious why the study only considered between IAV and RV, but not IBV. Especially considering the circulation of IBV is not negligible in some regions in the US (e.g., Region 6, 9, and 10 in 2015/16 season). The observed interference patterns and transmission dynamics could instead reflect the co-circulation of RV and different strains of influenza virus, together with their time-varying population immunity arising from prior infection, cross-immunity, and genetic changes in circulating strains.

Thank you for this comment. We only considered IAV because most prior epidemiological and experimental studies on viral interference with RV we found in the literature have either focused on IAV, or only found a negative association with IAV. However, we agree that we cannot rule out the possibility that IBV may interact with RV and we have thus performed additional analyses considering all IV detections, i.e., IAV+IBV (Fig. S23, and Results p 8, ln 248-251).

Referee: 3

The authors investigate potential interactions between Human Rhinovirus and Influenza from the analysis of long time series of surveillance from the US and Canada. They develop a mathematical modelling approach to investigate to which extent perturbations, such as those that occurred during the pandemic due to unprecedent interventions affecting the epidemiology and circulation of most respiratory viruses, can increase interactions identifiability. They find that indeed, perturbations make it possible to improve the estimation of interactions; estimating interactions that would not have been identified significantly in normal seasons. Applying the model to surveillance data from the US and Canada, they found globally no significant interactions, although an unexplained difference between the two countries was obtained. This is an important study that clearly contributes to previous literature related to interactions analysis using modelling. My concerns and comments on the methods, data and assumptions are detailed below.

Thank you very much for your positive and useful comments, as well as your work on our manuscript. We detail below how we took into account your comments in our revision.

Major comments:

- It was not clear to me how RV/EV was defined here. The manuscript indicates that the virus(es) was/were tested using PCRs which test for mixt rhinovirus and enteroviruses. Is it a global multiplex PCR? It would be good to provide a bit more precision. To which extent is this technique specific to Rhinovirus? Which virus are we talking about here? Do we expect all RV/EV viruses to have the same effect related to flu? More information on the viruses that are tested is important, together with their infection trends, and season. For example, it is stated that the pandemic did not affect the dynamics of RV, which has been described in many countries – is it the case for both RV and EV? How was this virus diversity accounted for in terms of dynamic infection in the population? My understanding is that the model is a global model of different viruses, what is the consequence of this in terms of assumptions or interpretations?

NREVSS (US) and RVDSS (Canada) are laboratory-based surveillance systems that monitor respiratory virus activity across the country (e.g., RSV, hMPV, HCOV, SARS-CoV-2, (para)influenza, adenoviruses). Importantly, surveillance data come from people with respiratory symptoms, which in part explains why RV is the dominant virus. You're right that weekly tests and detections mostly derived from multiplex PCR test results. Rapid laboratory diagnostic testing does not differentiate RVs from other EVs, and results are typically reported as a combined RV/EV category. While there is no strong evidence for viral interference between IAV and other EVs, RVs are expected to account for most positive detections (consistent with, e.g., (Monto et al. 2025)). For example, in a recent European study, RV was present in 45% of people with acute respiratory infections, while EV was found in only 3% (Vlaicu et al. 2026). We therefore considered RV/EV patterns to be primarily

driven by RV circulation, which is a classic assumption (e.g., (Monto et al. 2025), or for data from 2009 in (Nickbakhsh et al. 2019)). We have included and emphasized this point in Materials and Methods p 12, ln 423-432. The COVID-19 pandemic did affect RV circulation (especially during the very first implementation of NPIs), though much less than for most other respiratory pathogens and RV continued to circulate at appreciable levels in many countries (Introduction p 3, ln 88-91, where cited references include China, South Korea, Brazil, Austria, Germany, Switzerland, Italy, England, Finland and New Zealand, and mostly relied on RV/EV data as well). Other EVs usually peak more in the late summer and seem more affected by NPIs, such as EV D68 (Park et al. 2024a). We indeed modeled RVs as a single-strain pathogen, thereby neglecting the different RV species and underlying antigenic diversity (we discussed this point and the lack of data in Discussion p 11, ln 377-393). Consequences include that the seasonal transmission rates β represents an average transmission profile across co-circulating RV types (Discussion p 11, ln 378-382) and thus should be interpreted with care, and that the inferred short duration of immune protection is more likely to reflect the co-circulation of any subtypes with very weak cross-immunity than rapid waning of immunity (Discussion p 10, ln 327-329). Differences between countries (or even at the regional level) may also potentially reflect differences in strain composition (Discussion p 11, ln 380-382).

- What were the reporting rates of IAV and RV (in the real surveillance system and in the model)? More details on how the surveillance systems are set up in the countries, and possible evolutions over time (especially before, during the pandemic, and after) would be useful to get insights into their stability, and the considered population? For example, are the individuals infected by RV/EV the same as the ones by Flu? Who are the tested people?

Incidence data came from decade-long surveillance system established by the CDC and the Public Health Agency of Canada to monitor respiratory viruses. The system involves a network of participating laboratories across each country testing patients with respiratory symptoms in outpatient and inpatient settings. Testing is conducted by PCR; in recent years the use of multiplex PCR has become more frequent (e.g., https://www.cdc.gov/nrevss/php/dashboard/index.html). We detailed this in Materials and Methods p 12, ln 423-427. Testing patterns are reported in Fig. S4 (RV/EV) and Fig. S5 (IAV). The true reporting rates are unknown. Importantly, incidence time series were rescaled in order to capture long-term changes in surveillance (Materials and Methods p 12-13, ln 434-440). Such long-term changes, which we are already accounting for, are likely to reflect changes in surveillance practices over time. On the other hand, shorter-term changes are much harder to capture and interpret, partly because they reflect real changes in transmission (e.g., surveillance significantly decreased during the COVID-19 pandemic because transmission was reduced by NPIs). Estimated values of ρ (*reporting rate*) were reported in table S3 for each location, but we note in Results p 8, ln 262-263, that they cannot be interpreted quantitatively as $\hat{\rho}$ also compensate for the above-mentioned arbitrary rescaling of detections. We renamed ρ as an under-reporting *scaling factor* to nuance a bit the description of this parameter.

- It is not clear for me what the interpretation of ϕ is at the individual level, could the authors give a bit more details?

The viral interaction parameter ϕ quantifies the direction and intensity of the effect IAV has on RV force of infection, i.e., to which extent IAV activity forces RV transmission at the population level. If ϕ is positive, it means IAV facilitates RV transmission, if negative, it prevents RV transmission (potentially via immune interference), and if zero, there is no evidence of interaction. We agree it comes from a mechanism acting at the level of individual hosts, but since ϕ is a population-level estimate,

there is not necessarily a direct biological individual-level quantity. Here, our approach is intentionally phenomenological and our model does not aim to explicitly resolve within-host interactions. Instead, it provides an effective description of observed incidence dynamics and allows us to probe for epidemiological signatures consistent with altered transmission patterns that integrate both within-host viral interference (immunological) mechanisms and ecological mechanisms of interactions (Bhattacharyya et al. 2015). We emphasized this point in Results p 5, ln 144-151 and Discussion p 10, ln 352-360.

- What are the explanations for such differences between Canadian locations and American locations that could explain such differences in estimates? More discussion and exploration of hypotheses would be useful. Could that be related to some population drivers? Or surveillance? Other?

Whether these differences are meaningful is unclear and otherwise difficult to explain. The different surveillance systems and the usually earlier timing of IAV rebounds in US locations appear to be the two main differences between the two countries. It is however difficult to assess precisely their impact on the estimation process. These differences were obtained when fitting the model independently to each location. We explored how fitting the model simultaneously to all regions/provinces impacted the results by constraining ϕ to be shared across locations within a given country (US vs. Canada) and the rate of immune waning ω across all locations (Supporting information §S3). However, note that this approach assumes that relative differences in IAV incidence reflect true differences in viral circulation between locations rather than spatial heterogeneity in reporting effort. Estimates $\hat{\phi}$ are centered around 0 for both the US and Canada, with no difference in sign, although the Canadian estimate has a very wide CrI. The fact that these differences do not persist when fitting jointly across locations seems to suggest that they don't reflect robust country-level differences. We included this analysis in the revised manuscript (Results p 7, ln 239-248, and Fig. S21).

- With regards to comparison to other studies, it raises the question of 'to which extent does age matter?'. To which extent do we expect that heterogeneities across ages would impact the results. Indeed, some viruses may circulate more in one group than the other or have a different timing depending on the group, which has been described before for some respiratory viruses, in particular during the pandemic. How would that affect the results, and the identifiability? Is the surveillance data available across age groups?

Yes, we agree that this is an important consideration, notably for RVs, for which age-specific differences in circulation patterns have often been reported. Age stratification was not available for the surveillance data used in this study, but we agree that such heterogeneity may have an influence. It may contribute to residual variation that is not explicitly captured by the model, and the use of aggregated data (despite the advantage of larger sample sizes) may obscure some important age-dependent patterns. Addressing this question would require an extension of the current modeling framework, incorporating information/assumptions about age-specific contact patterns, together with age-stratified surveillance data (not available to us), and would therefore constitute a natural future direction. We have expanded our Discussion (p 11, ln 367-373) to further acknowledge the limitation that age-specific differences are not explicitly represented in our model and that future work incorporating age structure could evaluate how this could improve model fit and parameter estimation.

- I really liked the simulation study. How were the parameters chosen? What is their impact? Were some sensitivity analyses carried out over the fixed parameters in this simulation study? If not, some should be done to identify to which extent the results presented here depend on virus parameters.

Thank you. We have added more details on parameter values in the caption of Table S1 (and pointed to it in Material and Methods p 16, ln 546-547). Virus parameters are based on existing literature and/or to reproduce the observed epidemiological characteristics of RV and IAV. With these parameter values, IAV exhibits a single annual peak during winter, whereas RV shows two seasonal peaks in fall and spring. The basic reproduction number ($\mathcal{R}_0 = \beta/(\gamma + \mu)$) ranges between 1.53 and 2.17 for RV, and between 1.33 and 2.0 for IAV, which is consistent with (Spencer et al. 2022; Leung 2021; Yang et al. 2015). The mean duration of infectiousness is set to $1/\gamma_{\mathrm{IAV}}$ =5 days for IAV (Carrat et al. 2008; Spencer et al. 2022), and $1/\gamma$ =8 days for RV (Spencer et al. 2022). The mean duration of IAV immunity is set to $1/\omega_{\mathrm{IAV}}$ =40 weeks, reflecting temporary protection over approximately only one influenza season due to antigenic drift. The different main simulations explored different profiles of the seasonal transmission rate of RV. We now examine further the sensitivity of our results to the chosen values of other parameters: ω (the mean duration of RV immune protection), κ (the strength of NPIs on RV transmission), κ_{IAV} (the strength of NPIs on IAV transmission) and a_{IAV} (the amplitude of IAV seasonal forcing). Most estimates remained robust across the explored range of value (Results p 6, 188-190, and Fig. S13).

- Justify the choice of the lognormal distribution, what is the impact on the inference?

We now better justify our choice of the log-Normal distribution in the main text (Material and Methods p 15, ln 488-489 and ln 491-493). We relied on a log-Normal distribution because $C[t]$ (RV detection proxy) is a positive, continuous variable, and epidemiological reporting processes typically exhibit multiplicative (rather than additive) variability, making a Gaussian model on the log scale appropriate. This is similar to, e.g., (Park et al. 2024b). The log-Normal distribution implies homoscedasticity on the log scale; substantial overdispersion or heavy tails could otherwise lead to underestimated uncertainty or biased parameter estimates under extreme reporting variability. Poisson and negative binomial likelihoods are also commonly used in similar epidemiological settings, but require discrete case counts.

- Here, the authors made the choice to use directly IAV data from surveillance instead of modelling its transmission. Although it can be easily understood why this assumption was made from a modelling point of view, it should be discussed more. Especially, what is the expected impact of this hypothesis, and where do we expect it to affect results?

Indeed, relying on IAV surveillance data avoids explicitly modeling IAV transmission dynamics, for which we have identified the following implications. First, we used weekly IAV incidence as a proxy for IAV prevalence. This approximation is reasonable given that the mean duration of infectiousness for IAV is approximately one week, implying a close correspondence between incidence and prevalence at the temporal resolution of our model (Material and Methods p 15, ln 502-504). Second, this approach does not allow to jointly reconstructing the transmission dynamics of both pathogens. Last, empirical data contains important aspects of influenza inter-annual variability that are difficult to capture by any existing model. This includes antigenic evolution, competition between resident lineages, seeding/importation, and subtype interference. By using the empirical data, we are ensuring we get proper signal of IAV dynamics, even though we are losing a bit in terms of noise (but the data are very aggregated, sampling is large and we smoothed the data). In contrast to a two-pathogen model, our current approach thus preserves this empirical variability, which is noisier but may also be more informative for inferring its effect on RV dynamics. We now discuss these points more explicitly in Discussion p 10, ln 343-360, and we also now cite (Shrestha et al. 2013) that similarly used influenza time series as a covariate.

- A related question – if I understood well, IAV value was rescaled between 0 and -1. Is this rescaling done over all seasons, or at the season level? Does that mean that we remove the potential impact of epidemic size and differences across seasons?

We rescaled $C_{\text{IAV}}[t]$ between 0 and 1, which guarantees that the force of infection remains positive when enforcing a lower bound of -1 for ϕ. Specifically, for each location, we simply divide $C_{\text{IAV}}[t]$ by its maximum value over the entire study period (same approach for lagged incidences). As we used the global maximum across all seasons, it only rescaled the time series and preserved any differences in epidemic size or any other relative differences across seasons. We have clarified this point in the main text (Material and Methods p 15, ln 505-508).

- It was very interesting that the authors tested the effect of lagged IAV incidences. However, it is not clear for me why the authors considered the sum here. Have they considered looking at the lags independently? ie C_{-1} only or C_{-2} only? Which would model a delay in the effect and not a cumulative effect. Or otherwise weighting over the time lags? How would this affect the interpretation and estimations here?

Thank you for this comment. We only considered cumulative sums of lagged incidence assuming that all people infected during the past l weeks remain in a "protected class" over that entire window (i.e., a sustained effect). This follows the approach of (Howerton et al. 2025) and is intended to approximate varying durations of potential immune protection or reduced susceptibility. We clarified this point in the main text (Material and Methods p 15, ln 510-512). On the other hand, looking at the lags independently would only consider a short-lived response ($\approx \Delta t = 1$ week) and, as you said, would introduce a delay in the effect, which seems more difficult to justify from an immunological or ecological perspective. We agree that a weighted lag structure might provide a more realistic representation of waning protection over time, but it would also require specifying a decay profile, thereby introducing additional structural assumptions and increasing the complexity of the estimation. Furthermore, if the potential interaction is primarily driven by interferon responses, the relevant protection is expected to be both fast and short-lived, as pointed out by Reviewer #1 – e.g., resolved within days to at most a week, which would be captured using only current IAV incidence or a 1-week lag (Material and Methods p 15, ln 512-514).

- The number of MCMC chains of 4000 updates seems quite small for the number and complexity of estimated parameters. How was the convergence? Can the authors provide the chains and posterior in supplement?

We now provide some representative example chains and corresponding posteriors in the supporting information (Fig. S16). Due to their volume, only a small representative subset is shown. As mentioned in the main text (Materials and Methods p 16, ln 527-530), we assessed the convergence of all posterior distributions by the absence of warnings from Stan, including no divergent transitions, sufficient maximum tree depth, Gelman-Rubin statistics (R-hat) below 1.05 and effective sample size (ESS) above 400 ($100 \times$ number of chains) for each parameter.

- Simulated detections were assumed to follow a lognormal with 0.25 sd. What is the impact of such assumption on the results? and CIs?

We assessed the impact of using a higher sd of observation error σ. We regenerated simulated data

using $\sigma = 0.5$ for the 3 main simulations and displayed estimated results in Fig. S12. Our results were robust to observation noise (Results p 6, ln 187-188).

- The details on the wavelet analysis should be provided in supplement, with equations provided. If was not clear to me how this analysis was used in the following. Might be good to clarify for the readers.

Cross-wavelet analysis was used as an exploratory tool to examine relationships in time-frequency space between RV/EV and IAV detections over time. The results were not used in subsequent statistical models but were included to confirm and complement the initial visualization and description of the time series, further motivating the subsequent analysis. We have clarified this point in the main text (Results p 4, ln 122-123) and provided more details on this analysis in the supporting information §S1.

Minor comments

- Fig1: Legend of colors is missing for the figure on the bottom

Added.

References

Bhattacharyya, Samit et al. (2015). "Cross-immunity between strains explains the dynamical pattern of paramyxoviruses". In: *Proceedings of the National Academy of Sciences* 112.43, pp. 13396–13400. DOI: 10.1073/pnas.1516698112.

Carrat, Fabrice et al. (2008). "Time lines of infection and disease in human influenza: a review of volunteer challenge studies". In: *American journal of epidemiology* 167.7, pp. 775–785. DOI: 10.1093/aje/kwm375.

Howerton, Emily et al. (2025). "Using COVID-19 pandemic perturbation to model RSV-hMPV interactions and potential implications under RSV interventions". In: *Nature communications* 16.1, p. 7261. DOI: 10.1038/s41467-025-62358-w.

Leung, Nancy HL (2021). "Transmissibility and transmission of respiratory viruses". In: *Nature Reviews Microbiology* 19.8, pp. 528–545. DOI: 10.1038/s41579-021-00535-6.

Monto, Arnold S et al. (2025). "Respiratory viral infections from 2015 to 2022 in the HIVE cohort of American households: incidence, illness characteristics, and seasonality". In: *The Journal of Infectious Diseases* 231.3, pp. 795–804. DOI: 10.1093/infdis/jiae423.

Nickbakhsh, Sema et al. (2019). "Virus–virus interactions impact the population dynamics of influenza and the common cold". In: *Proceedings of the National Academy of Sciences* 116.52, pp. 27142–27150. DOI: 10.1073/pnas.1911083116.

Park, Sang Woo et al. (2024a). "Predicting the impact of COVID-19 non-pharmaceutical intervention on short-and medium-term dynamics of enterovirus D68 in the US". In: *Epidemics* 46, p. 100736.

Park, Sang Woo et al. (2024b). "Predicting the impact of non-pharmaceutical interventions against COVID-19 on Mycoplasma pneumoniae in the United States". In: *Epidemics* 49, p. 100808. DOI: 10.1016/j.epidem.2024.100808.

Shrestha, Sourya et al. (2013). "Identifying the interaction between influenza and pneumococcal pneumonia using incidence data". In: *Science translational medicine* 5.191, 191ra84–191ra84. DOI: 10.1126/scitranslmed.3005982.

Spencer, Julie A et al. (2022). "Distinguishing viruses responsible for influenza-like illness". In: *Journal of theoretical biology* 545, p. 111145. DOI: 10.1016/j.jtbi.2022.111145.

Vlaicu, Ovidiu et al. (2026). "Epidemiology and clinical outcomes of common respiratory viruses detected by multiplex RT-PCR in patients at a national infectious disease reference center in Romania, 2022–2025". In: *Virology Journal*. DOI: 10.1186/s12985-026-03217-y.

Yang, Wan et al. (2015). "Forecasting influenza epidemics in Hong Kong". In: *PLoS computational biology* 11.7, e1004383. DOI: 10.1371/journal.pcbi.1004383.
