# Supplementary material for: Leveraging perturbations to infer the population dynamics of human rhinovirus and interaction of influenza A virus
Source: PLoS Comput Biol. 2026 Sep 11;22(9):e1014784. doi: 10.1371/journal.pcbi.1014784 (PMC13585339; doi:10.1371/journal.pcbi.1014784)
Supplement: Peer review history

# Peer review: Decision Letter 0

**DOI:** 10.1371/journal.pcbi.1014784.r001

**Authors:** Thomas Leitner, Michael Plank

17 Jun 2026

Leveraging perturbations to infer the population dynamics of human rhinovirus and interaction of influenza A virus

PLOS Computational Biology

Dear Dr. Benhamou,

Thank you for submitting your manuscript to PLOS Computational Biology. After careful consideration, we feel that it has merit but does not fully meet PLOS Computational Biology's publication criteria as it currently stands. Therefore, we invite you to submit a revised version of the manuscript that addresses the points raised during the review process.

Please submit your revised manuscript by Aug 17 2026 11:59PM. If you will need more time than this to complete your revisions, please reply to this message or contact the journal office at ploscompbiol@plos.org. When you're ready to submit your revision, log on to https://www.editorialmanager.com/pcompbiol/ and select the 'Submissions Needing Revision' folder to locate your manuscript file.

Please include the following items when submitting your revised manuscript:

* A letter that responds to each point raised by the editor and reviewer(s). You should upload this letter as a separate file labeled 'Response to Reviewers'. This file does not need to include responses to formatting updates and technical items listed in the 'Journal Requirements' section below.

* A marked-up copy of your manuscript that highlights changes made to the original version. You should upload this as a separate file labeled 'Revised Manuscript with Track Changes'.

* An unmarked version of your revised paper without tracked changes. You should upload this as a separate file labeled 'Manuscript'.

We look forward to receiving your revised manuscript.

Kind regards,

Michael J Plank

Academic Editor

PLOS Computational Biology

Thomas Leitner

Section Editor

PLOS Computational Biology

**Additional Editor Comments (if provided):**

AE comments:

The three reviewers agree that the paper makes a strong methodological contribution, but identify a number of areas where the implementation, justification of modelling assumptions, and writing need improvement. Please ensure you address all reviewer comments in a revised version.

**Journal Requirements:**

If the reviewer comments include a recommendation to cite specific previously published works, please review and evaluate these publications to determine whether they are relevant and should be cited. There is no requirement to cite these works unless the editor has indicated otherwise.

1) Please ensure that the CRediT author contributions listed for every co-author are completed accurately and in full.

At this stage, the following Authors/Authors require contributions: Wakinyan Benhamou, Emily Howerton, Sang Woo Park, Cécile Viboud, C Jessica E Metcalf, and Bryan Thomas Grenfell. Please ensure that the full contributions of each author are acknowledged in the "Add/Edit/Remove Authors" section of our submission form.

The list of CRediT author contributions may be found here: https://journals.plos.org/ploscompbiol/s/authorship#loc-author-contributions

2) We ask that a manuscript source file is provided at Revision. Please upload your manuscript file as a .doc, .docx, .rtf or .tex. If you are providing a .tex file, please upload it under the item type u2018LaTeX Source Fileu2019 and leave your .pdf version as the item type u2018Manuscriptu2019.

3) Please provide an Author Summary. This should appear in your manuscript between the Abstract (if applicable) and the Introduction, and should be 150-200 words long. The aim should be to make your findings accessible to a wide audience that includes both scientists and non-scientists. Sample summaries can be found on our website under Submission Guidelines:

https://journals.plos.org/ploscompbiol/s/submission-guidelines#loc-parts-of-a-submission

4) Please upload all main figures as separate Figure files in .tif or .eps format. For more information about how to convert and format your figure files please see our guidelines:

https://journals.plos.org/ploscompbiol/s/figures

5) Please upload a copy of Figure Fig 1-5 which you refer to in your text on pages 4, 5, 6, and 7. Or, if the figure is no longer to be included as part of the submission please remove all reference to it within the text.

6) Please amend your detailed Financial Disclosure statement. This is published with the article. It must therefore be completed in full sentences and contain the exact wording you wish to be published.

i) State what role the funders took in the study. If the funders had no role in your study, please state: "The funders had no role in study design, data collection and analysis, decision to publish, or preparation of the manuscript."

ii) If any authors received a salary from any of your funders, please state which authors and which funders..

If you did not receive any funding for this study, please simply state: u201cThe authors received no specific funding for this work.u201d

7) We have amended your Competing Interest statement to comply with journal style. We kindly ask that you double check the statement and let us know if anything is incorrect.

**Reviewers' comments:**

Reviewer's Responses to Questions

**Comments to the Authors:**

**Please note here if the review is uploaded as an attachment.**

Reviewer #1: the review is uploaded as an attachment.

Reviewer #2: Thank you for the opportunity to review the manuscript. This work investigates the population-level interactions between respiratory viruses using long-term surveillance data and mechanistic modeling approaches. The authors demonstrate that exogenous epidemiological perturbations, such as non-pharmaceutical interventions, can help disentangle pathogen–pathogen interactions from confounding seasonal effects. In particular, the study finds no conclusive evidence that influenza A virus substantially alters rhinovirus dynamics at the population level. Overall, the work addresses an interesting question in infectious disease epidemiology and contributes to the understanding of respiratory virus interactions and seasonality. However, I do have the following concerns.

1. I am therefore not fully convinced that the current framework is sufficient to assess viral interference. Conceptually, viral interference refers to interactions between viruses within a host that alter the infection outcome of at least one virus at the individual level. These within-host interactions subsequently shape population-level transmission dynamics and should be evaluated while accounting for key population characteristics, including immunity and co-circulation patterns. In this context, a multi-strain modeling framework may provide a more appropriate approach to address the research questions.

2. In the introduction, the hypotheses were clearly laid out for the bi-directional viral interference between RV and influenza. However, the model authors only considered viral interference from IAV to RV.

3. I am curious why the study only considered between IAV and RV, but not IBV. Especially considering the circulation of IBV is not negligible in some regions in the US (e.g., Region 6, 9, and 10 in 2015/16 season). The observed interference patterns and transmission dynamics could instead reflect the co-circulation of RV and different strains of influenza virus, together with their time-varying population immunity arising from prior infection, cross-immunity, and genetic changes in circulating strains.

Reviewer #3: The authors investigate potential interactions between Human Rhinovirus and Influenza from the analysis of long time series of surveillance from the US and Canada. They develop a mathematical modelling approach to investigate to which extent perturbations, such as those that occurred during the pandemic due to unprecedent interventions affecting the epidemiology and circulation of most respiratory viruses, can increase interactions identifiability. They find that indeed, perturbations make it possible to improve the estimation of interactions; estimating interactions that would not have been identified significantly in normal seasons. Applying the model to surveillance data from the US and Canada, they found globally no significant interactions, although an unexplained difference between the two countries was obtained. This is an important study that clearly contributes to previous literature related to interactions analysis using modelling. My concerns and comments on the methods, data and assumptions are detailed below.

Major comments:

- It was not clear to me how RV/EV was defined here. The manuscript indicates that the virus(es) was/were tested using PCRs which test for mixt rhinovirus and enteroviruses. Is it a global multiplex PCR? It would be good to provide a bit more precision. To which extent is this technique specific to Rhinovirus? Which virus are we talking about here? Do we expect all RV/EV viruses to have the same effect related to flu? More information on the viruses that are tested is important, together with their infection trends, and season. For example, it is stated that the pandemic did not affect the dynamics of RV, which has been described in many countries – is it the case for both RV and EV? How was this virus diversity accounted for in terms of dynamic infection in the population? My understanding is that the model is a global model of different viruses, what is the consequence of this in terms of assumptions or interpretations?

- What were the reporting rates of IAV and RV (in the real surveillance system and in the model)? More details on how the surveillance systems are set up in the countries, and possible evolutions over time (especially before, during the pandemic, and after) would be useful to get insights into their stability, and the considered population? For example, are the individuals infected by RV/EV the same as the ones by Flu? Who are the tested people?

- It is not clear for me what the interpretation of Phi is at the individual level, could the authors give a bit more details?

- What are the explanations for such differences between Canadian locations and American locations that could explain such differences in estimates? More discussion and exploration of hypotheses would be useful. Could that be related to some population drivers? Or surveillance? Other?

- With regards to comparison to other studies, it raises the question of ‘to which extent does age matter?’. To which extent do we expect that heterogeneities across ages would impact the results. Indeed, some viruses may circulate more in one group than the other or have a different timing depending on the group, which has been described before for some respiratory viruses, in particular during the pandemic. How would that affect the results, and the identifiability? Is the surveillance data available across age groups?

- I really liked the simulation study. How were the parameters chosen? What is their impact? Were some sensitivity analyses carried out over the fixed parameters in this simulation study? If not, some should be done to identify to which extent the results presented here depend on virus parameters.

- Justify the choice of the lognormal distribution, what is the impact on the inference?

- Here, the authors made the choice to use directly IAV data from surveillance instead of modelling its transmission. Although it can be easily understood why this assumption was made from a modelling point of view, it should be discussed more. Especially, what is the expected impact of this hypothesis, and where do we expect it to affect results?

- A related question – if I understood well, IAV value was rescaled between 0 and -1. Is this rescaling done over all seasons, or at the season level? Does that mean that we remove the potential impact of epidemic size and differences across seasons?

- It was very interesting that the authors tested the effect of lagged IAV incidences. However, it is not clear for me why the authors considered the sum here. Have they considered looking at the lags independently? ie C_-1 only or C_-2 only? Which would model a delay in the effect and not a cumulative effect. Or otherwise weighting over the time lags? How would this affect the interpretation and estimations here?

- The number of MCMC chains of 4000 updates seems quite small for the number and complexity of estimated parameters. How was the convergence? Can the authors provide the chains and posterior in supplement?

- Simulated detections were assumed to follow a lognomal with 0.25 sd. What is the impact of such assumption on the results? and CIs?

- The details on the wavelet analysis should be provided in supplement, with equations provided. If was not clear to me how this analysis was used in the following. Might be good to clarify for the readers.

Minor comments

- Fig1:

o Legend of colors is missing for the figure on the bottom

**********

**Have the authors made all data and (if applicable) computational code underlying the findings in their manuscript fully available?**

The PLOS Data policy requires authors to make all data and code underlying the findings described in their manuscript fully available without restriction, with rare exception (please refer to the Data Availability Statement in the manuscript PDF file). The data and code should be provided as part of the manuscript or its supporting information, or deposited to a public repository. For example, in addition to summary statistics, the data points behind means, medians and variance measures should be available. If there are restrictions on publicly sharing data or code —e.g. participant privacy or use of data from a third party—those must be specified.

Reviewer #1: None

Reviewer #2: None

Reviewer #3: Yes

**********

PLOS authors have the option to publish the peer review history of their article (what does this mean?). If published, this will include your full peer review and any attached files.

If you choose “no”, your identity will remain anonymous but your review may still be made public.

**Do you want your identity to be public for this peer review?**  For information about this choice, including consent withdrawal, please see our Privacy Policy.

Reviewer #1: **Yes:** Fanny Bergström

Reviewer #2: No

Reviewer #3: No

[NOTE: If reviewer comments were submitted as an attachment file, they will be attached to this email and accessible via the submission site. Please log into your account, locate the manuscript record, and check for the action link "View Attachments". If this link does not appear, there are no attachment files.]

**Figure resubmission:**

**Reproducibility:**

?>

Attachment Submitted filename: review_plos.pdf

# Author comment: Author response to Decision Letter 1

**DOI:** 10.1371/journal.pcbi.1014784.r002

4 Aug 2026

Attachment Submitted filename: Response_to_Reviewers.pdf

# Peer review: Decision Letter 1

**DOI:** 10.1371/journal.pcbi.1014784.r003

**Authors:** Thomas Leitner, Michael Plank

1 Sep 2026

Dear M Benhamou,

We are pleased to inform you that your manuscript 'Leveraging perturbations to infer the population dynamics of human rhinovirus and interaction of influenza A virus' has been provisionally accepted for publication in PLOS Computational Biology.

Before your manuscript can be formally accepted you will need to complete some formatting changes, which you will receive in a follow up email. A member of our team will be in touch with a set of requests.

Please note that your manuscript will not be scheduled for publication until you have made the required changes, so a swift response is appreciated.

IMPORTANT: The editorial review process is now complete. PLOS will only permit corrections to spelling, formatting or significant scientific errors from this point onwards. Requests for major changes, or any which affect the scientific understanding of your work, will cause delays to the publication date of your manuscript.

Should you, your institution's press office or the journal office choose to press release your paper, you will automatically be opted out of early publication. We ask that you notify us now if you or your institution is planning to press release the article. All press must be co-ordinated with PLOS.

Thank you again for supporting Open Access publishing; we are looking forward to publishing your work in PLOS Computational Biology.

Best regards,

Michael J Plank

Academic Editor

PLOS Computational Biology

Thomas Leitner

Section Editor

PLOS Computational Biology

***********************************************************

Reviewer's Responses to Questions

**Comments to the Authors:**

**Please note here if the review is uploaded as an attachment.**

Reviewer #1: The authors have satisfactorily addressed all the concerns raised in my previous review. I have no further comments and recommend the manuscript be accepted for publication. I thank the authors and editor for the opportunity to review this work.

Reviewer #2: The authors have clearly addressed all of my concerns. I have no further questions.

**********

**Have the authors made all data and (if applicable) computational code underlying the findings in their manuscript fully available?**

The PLOS Data policy requires authors to make all data and code underlying the findings described in their manuscript fully available without restriction, with rare exception (please refer to the Data Availability Statement in the manuscript PDF file). The data and code should be provided as part of the manuscript or its supporting information, or deposited to a public repository. For example, in addition to summary statistics, the data points behind means, medians and variance measures should be available. If there are restrictions on publicly sharing data or code —e.g. participant privacy or use of data from a third party—those must be specified.

Reviewer #1: Yes

Reviewer #2: None

**********

PLOS authors have the option to publish the peer review history of their article (what does this mean?). If published, this will include your full peer review and any attached files.

If you choose “no”, your identity will remain anonymous but your review may still be made public.

**Do you want your identity to be public for this peer review?**  For information about this choice, including consent withdrawal, please see our Privacy Policy.

Reviewer #1: **Yes:** Fanny Bergström

Reviewer #2: No

# Editor report: Acceptance letter

**DOI:** 10.1371/journal.pcbi.1014784.r004

**Authors:** Thomas Leitner, Michael Plank

PCOMPBIOL-D-26-00664R1

Leveraging perturbations to infer the population dynamics of human rhinovirus and interaction of influenza A virus

Dear Dr Benhamou,

I am pleased to inform you that your manuscript has been formally accepted for publication in PLOS Computational Biology. Your manuscript is now with our production department and you will be notified of the publication date in due course.

The corresponding author will soon be receiving a typeset proof for review, to ensure errors have not been introduced during production. Please review the PDF proof of your manuscript carefully, as this is the last chance to correct any errors. Please note that major changes, or those which affect the scientific understanding of the work, will likely cause delays to the publication date of your manuscript.

Soon after your final files are uploaded, unless you have opted out, the early version of your manuscript will be published online. The date of the early version will be your article's publication date. The final article will be published to the same URL, and all versions of the paper will be accessible to readers.

For Research, Software, and Methods articles, you will receive an invoice from PLOS for your publication fee after your manuscript has reached the completed accept phase. If you receive an email requesting payment before acceptance or for any other service, this may be a phishing scheme. Learn how to identify phishing emails and protect your accounts at https://explore.plos.org/phishing.

Thank you again for supporting PLOS Computational Biology and open-access publishing. We are looking forward to publishing your work!

With kind regards,

Janani Seenivasan

PLOS Computational Biology | Carlyle House, Carlyle Road, Cambridge CB4 3DN | United Kingdom ploscompbiol@plos.org | Phone +44 (0) 1223-442824 | ploscompbiol.org | @PLOSCompBiol
